# Therapists’ perceptions of alliance barriers in the parent–therapist relationship when treating children who have experienced trauma

**DOI:** 10.1080/00049530.2025.2528346

**Published:** 2025-07-17

**Authors:** Simene Joffe, Veronica M Dwarika

**Affiliations:** Department of Educational Psychology, Faculty of Education, University of Johannesburg, Johannesburg, South Africa

**Keywords:** Child trauma, parent–therapist alliance, parent work, therapeutic alliance, trauma

## Abstract

**Objective:**

Childhood experiences of traumatic events are common in all countries, and children may require psychotherapy after a traumatic experience. For those children who have experienced trauma and are involved in therapy, the parent–therapist relationship is important in promoting child trauma recovery. However, alliance barriers can interrupt the working relationship, compromising child treatment efficacy. Few studies have explored therapists’ perceptions of the complexities in the parent–therapist relationship when treating children for trauma.

**Method:**

This exploratory study uses trauma theory to understand obstacles interfering with the parent–therapist alliance. Qualitative research methods were used to explore data obtained from 15 therapists. An interpretive phenomenological research design supported the study to answer the research question.

**Results:**

Therapists were motivated to work with parents, as they recognise the important role of parents in treatment. They identified alliance barriers as reduced parent investment, parent trauma and therapists’ emotions. Therapists also applied a trauma-informed framework to moderate alliance obstacles preventing them from impacting child treatment success.

**Conclusion:**

The findings highlight that therapists require knowledge of trauma theory and expertise in trauma-informed practice to work effectively with parents when treating children for trauma. This new knowledge will help therapists manage and support the parent–therapist relationship when treating children who have experienced trauma, improving long-term treatment outcomes of child psychotherapy.

## KEY POINTS

**What is already known about this topic:**

1. Childhood experiences of trauma are universal, and children may require psychotherapy treatment.
2. Evidence-based literature proves trauma-focused interventions are effective in treating child trauma, yet child attrition rates continue to be high.
3. Positive parent–therapist alliances improve child psychotherapy success.

**What this topic adds:**

1. This exploratory research study identifies alliance barriers in the parent–therapist relationship when working with parents of children who have experienced trauma.
2. The research provides practitioners with new information on the complexities and tensions occurring in the parent–therapist relationship when treating children after traumatic experiences.
3. Therapists can use this new understanding to better manage and support parents in the working alliance to improve child retention rates and outcomes.

**Preliminary recommendations:**

1. Child psychotherapists need knowledge and expertise in trauma-specific practices to work effectively with parents when treating children who have experienced trauma.
2. Future research can compile guidelines for therapists on how to work more effectively with parent trauma survivors.

## Introduction

A significant number of children experience traumatic events in their lifetime. Trauma is a bipartite concept which includes potential traumatic exposure to an event/s causing psychological distress, and the individual’s subjective response to the event/s (actual reactions). In this paper, potential traumatic exposure is defined as encountering a life-threatening event, and trauma experience refers to the psychological harm resulting from experiencing or witnessing the event. Furthermore, traumatic experiences stem from a variety of circumstances (inter-familial, effects of poverty, war experiences, etc.). Whether single or multiple traumatic experiences, the effects of emotional trauma range from minimal to considerable, manifest differently within and between people and vary over time. While trauma reactions are not always clinical in nature or severity, a considerable proportion of the population has experienced traumatic incidents, leaving them vulnerable to the lifelong consequences of trauma.

Psychotherapy can be effective in reducing the impact of the trauma to help children live more meaningful lives (Downey & Crummy, 2022; Landolt & Kenardy, 2022), yet child attendance rates continue to be low and attrition rates high. Given that a strong parent–therapist relationship promotes parental investment, cooperation and treatment support to improve child attendance rates and outcomes (Marks, 2020), the therapist’s management of the parent–therapist relationship is key to the success of child psychotherapy. Although therapeutic interventions vary significantly in theoretical underpinning, approach, duration and the nature of the parent role (Substance Abuse and Mental Health Services Administration, 2016), when the parent–therapist relationship is characterised by mutual respect, collaboration and egalitarianism, it can be a mechanism of change progressing parent functioning and capacity to advance child trauma recovery. To date, there is little information on therapists’ perceptions of alliance obstacles when engaged in parent work when treating children who have experienced trauma. This paper offers a new perspective on the lived experience of therapists regarding the challenges they face in containing, managing and co-operating with parents when treating children’s trauma in psychotherapy within a trauma-informed paradigm.

In clinical work with parents, parents and psychotherapists place the child at the centre of treatment, to empower the child and create space for processing and understanding feelings (Holmes, 2018; Marks, 2020). They work collaboratively in supporting the child to achieve optimal therapeutic results. However, alliance tensions, barriers and obstacles can occur during parent–therapist interactions, disrupting the formation of a positive relationship. Examples are parent resistance concerns, non-compliance or treatment interference problems which obstruct child treatment outcome, consequently resulting in cancelled appointments, non-attendance and early termination of treatment (Pitillas, 2020). The literature corroborates that therapists encounter various forms of parental resistance behaviours in child psychotherapy, such as refusal to participate, denial of the child’s problem and hostility/defensiveness (Pitillas, 2020). Furthermore, parental trauma can manifest as difficult behaviours (dysregulation, extreme/inappropriate behaviours) and can significantly impact the quality of the parent – therapist relationship. Trauma theory argues that challenging behaviours are a direct result of a traumatic experience, which increases vulnerability to neurobiological changes, mental health disorders and levels of epistemic trust. To build positive relationships with trauma survivors, therapists need to adopt a trauma-informed care approach. This allows therapists to perceive parent-resistant behaviours in the context of trauma, understand that trauma responses drive maladaptive and treatment-oppositional behaviours and respond appropriately to parent trauma needs.

The main findings of previous empirical studies regarding the parent – therapist relationship highlight that alliance tensions and obstacles are concerning for therapists (Brown, 2020; Marks, 2020), since they decrease parent investment in child treatment, obstruct treatment objectives and prevent the formation of a robust parent – therapist relationship, subsequently compromising child treatment success

The study was underpinned by a trauma theoretical framework, since the research explored the relationship between parent and therapist to identify obstacles and tensions when treating children who have experienced trauma. This framework is crucial in the treatment and management of complex therapeutic problems. First, it creates a framework for therapists in the treatment of clients (children and parents) presenting with complex trauma [prolonged and repeated trauma] (Siverns & Morgan, 2019), and second, it provides strategies for therapists to recognise their own history of exposure to traumatic experiences and prevent secondary trauma. Therapists’ knowledge and understanding of trauma theory and trauma-informed care ensure that therapists have the expertise to manage and support parents effectively.

## Objectives

The parent–therapist relationship can expedite and sustain child trauma recovery. However, alliance barriers can interfere with the development of a robust working alliance. This study focused on exploring therapist perceptions of working collaboratively in therapy with parents of children who have experienced trauma. The aim was to gain an understanding of the complexities and tensions within the parent–therapist relationship. This new knowledge will assist therapists in better managing and supporting the working alliance, improving child retention rates and outcomes.

## Method

### Participants and recruitment

Fifteen therapists were purposefully selected. They included educational psychologists, clinical psychologists and registered counsellors recruited from private practitioners, United Relationships (pseudonym), Western Australia (WA) and community psychologists in Western Australia as shown in Tables 1 and 2. Pseudonyms were used for all therapists and organisations to protect confidentiality. Inclusion criteria included therapists having a minimum of 1 year’s work experience, being registered with a governing professional body and working in the public/private sector, in Perth, Western Australia. Ethical approval for this study was obtained through the University of Johannesburg.

**Table 1.** Biographical data of therapists and agency.

Therapists/agency	Age	Gender	Role	Years in practice	Work: Public or private sector
Participant 1 Charlotte	36–60	Female	Counsellor	12 years	Public
Participant 2 Katherine	36–60	Female	Counsellor	1 year	Private
Participant 3 Reese	36–60	Female	Psychologist	10 years	Private
Participant 4 Bailey	36–60	Female	Psychologist	18 years	Private
Participant 5 Nicole	18–35	Female	Psychologist	10 years	Private
Participant 6 Francis	61–65	Male	Psychologist	25 years	Private
Participant 7 Gianna	36–60	Female	Psychologist	10 years	Both
Participant 8 Amelia	18–35	Female	Counsellor	2 years	Both
Participant 9 Jackie	36–60	Female	Counsellor	1 year	Private
Participant 10 Gavin	36–60	Male	Psychologist	32 years	Private
Participant 11 Maya	36–60	Female	Counsellor	10 years	Both
Participant 12 Blake	36–60	Male	Psychologist	8 years	Private
Participant 13 Kate	36–60	Female	Counsellor	4 years	Private
Participant 14 Rowan	36–60	Female	Psychologist	22 years	Both
Participant 15 Tahlia	36–60	Female	Psychologist	31 years	Both
Agency-United Relationships	Not-for-profit organisation				Public service

**Table 2.** Summary of therapists.

Mean age	46.13 years	
Gender distribution	Females 80%	Males 20%
Mean years of experience	13.07 years	

### Procedure

After receiving ethics clearance from the University of Johannesburg (Approval number: Sem 1–2023–004), a qualitative study was conducted using convenience sampling. This type of sampling provided an in-depth and comprehensive exploration of the research phenomenon to answer the research question. Therapists completed an open-ended questionnaire, which was emailed to them by the researchers or distributed to them by the manager/psychologist, if affiliated with the agency/community. All therapists gave voluntary consent prior to participation and agreed to the privacy terms and data collection use. Regarding risk, all therapists had mandatory supervision and organisational support. If the research raised any personal concerns, therapists could contact a clinical psychologist for a one-off debrief and containment appointment or email the researchers, who would refer them to an alternative support/debrief service. None of the therapists made a “request” for a debriefing.

### Data collection

An open-ended questionnaire and reflective journal were the primary data collection methods used. The first research tool was an open-ended questionnaire, which all therapists completed. Researchers minimised cultural bias through awareness, checking and identifying potential bias in both questionnaire design and questions to ensure fairness (Choi & Pak, 2004). The questionnaire included closed- and open-ended questions. The first question related to demographic details and ensured that therapists fit the research criteria. The second focused on exploring therapists’ “lived experiences” within the parent – therapist alliance. An open-ended questionnaire was selected for two purposes. First, the data collection process required a tool where the researchers did not have any face-to-face contact with the therapists to protect client privacy, and second, open-ended questions encouraged therapists to freely express perspectives and personal experiences in their own words, facilitating fairness. The overall advantage of the research questionnaire was that it allowed therapists to respond privately and anonymously.

The researchers also maintained a self-reflective journal. Weekly entries were written to record and examine views, values and feelings throughout the research process. This assisted in counteracting any potential bias in the qualitative study (Ortlipp, 2008).

### Analysis

The research study adopted an interpretive qualitative approach and used qualitative methods to analyse the data. Creswell’s (2007) model of qualitative data analysis, with aspects of Braun and Clarke’s (2006) reflexive thematic analysis method, was applied to analyse the data. As Braun and Clarke’s (2006) inductive coding strategy was applied to the research, codes were derived directly from the data, and themes emerged organically from the information. Exploring the parent–therapist relationship, while treating children’s trauma, evolved through an iterative research process to produce new knowledge (Creswell & Poth, 2023) about how to better support and manage the working relationship.

### Reporting

The COREQ statement, a quality checklist for qualitative studies, was applied to ensure all relevant aspects of the research had been reported (Tong et al., 2007). However, not all questions were applicable since it was designed for interviews/focus groups.

## Results

Data analysis from the research question on examining therapists’ perceptions of working with parents whose children had experienced trauma revealed four key themes, shown in Table 3: parent investment in trauma-informed child psychotherapy treatment; therapists’ perspectives on parents’ past trauma; therapists emotional reactions; and addressing barriers within the parent–therapist relationship. These will be explored in detail in the section below (pseudonyms were used for all therapists quoted in this section).

**Table 3.** Themes.

1	Parent Investment in Trauma-informed Psychotherapy Treatment
2	Therapists’ Perspectives on Parents’ Past Trauma
3	Therapists’ Emotional Reactions
4	Addressing Barriers Within the Parent–Therapist Relationship

### Theme 1: parent investment in trauma-informed psychotherapy treatment

The first theme emerging from the data as an alliance barrier was reduced parent investment in child psychotherapy treatment. Therapists believed parents are important in child psychotherapy trauma treatment, as they enable child treatment attendance and effectuate change within the system. However, therapists identified several factors impacting parent investment in child psychotherapy treatment.

#### Parent knowledge and understanding of trauma

All 15 therapists felt parents have insufficient knowledge and understanding of trauma, which obstructs parent investment and support of child treatment. Therapists expressed that parents are unable to recognise the signs of trauma and are unaware that the child has experienced trauma. For example, Therapist Charlotte said the parents “*medicate the child, instead of recognising their young person has trauma”*. Other therapists added that parents *“minimise the impact of trauma”* (Therapist Tahlia) or “*deny there’s an issue”* (Therapist Gianna). These factors impact parents’ motivation to access and invest in trauma-informed psychotherapy services for their children.

#### Parent mentalisation of the child in the psychotherapy process

Twelve therapists indicated that parents’ decreased mentalisation capacity is another alliance barrier to parent investment in child treatment. Mentalisation ability is a mental activity that parents use to identify, interpret and understand the child’s thoughts and actions. Parent mentalisation of the child is important in trauma-focused therapy, since it fosters parental empathy, facilitates parental emotional regulation and increases a parent’s ability to predict and accommodate the child’s psychological needs (Fonagy et al., 2018). In this study, most therapists found that parents are *“unable to mentalise their child … . unable to hold onto their own thoughts and pause to let the child tell their story”* (Therapist Gianna). Therapists observed other signs which reveal parents’ difficulty in mentalising their child (*can’t understand the emotions of the child, you need to see behind your child’s behaviour*). They pointed out that parents with trauma histories have diminished parenting ability and react to the child with hostility or engage in undesirable parenting behaviours (*parent neglect and abuse behaviours, damages/hurts your child*). Essentially, the lessened parent mentalisation capacity impedes the child’s emotional recovery and creates tension within the working alliance.

#### Parent identification of the child and trauma behaviours

An overall concern of therapists was parents’ “*attitude towards the child, e.g., blaming the child and their behaviour, seeing the child as the problem”* (Therapist Reese), as in these circumstances, parents are less invested in child treatment. Two-thirds of therapists suggested parents blame the child for the trauma behaviours (*disrespectful, acting out behaviours, behaviours cause too much harm*) and consequently identify the child as the “problem” (*seeing the child as the problem, the child is the problem*). These parental behaviours interfere with the working alliance, given that parents are unable to see beyond the child’s behaviour to focus on the treatment goals and/or they expect the child to take ownership and “fix” the problem. In these circumstances, parents become less supportive of child treatment, hindering the parent–therapist alliance.

#### Parental responsibility and contribution to the child’s trauma responses

The findings showed parents are disinclined to take responsibility for their responses to their children’s trauma presentations. Most therapists described parents as unwilling to explore their own behaviours and are “*not taking ownership of their contribution*” (Therapist Katherine) to the child’s trauma response. Therapists also noted parents react with “*harshness, anxiety, anger and shame and fear*”. These parental behaviours impact child trauma healing and interfere with treatment goals, impeding the parent – therapist alliance.

However, therapists pointed out that most parents, in this study, “*have been through trauma themselves*” (Therapist Amelia, Therapist Reese, Therapist Gavin) and in some cases, the child’s trauma experience “*activate[s] … parents’ trauma history”* (Therapist Reese). This finding indicates that trauma compromises parents’ ability to acknowledge and agree to take responsibility (open, willing, engaged, cooperative) for the problem. This lack of parental ownership of the problem (child’s trauma) generates an added alliance barrier, as parents are less willing to take responsibility and enter into a therapeutic alliance.

#### Parental inclusion in treatment enhances parental investment

Therapists perceived a decrease in parental investment as an alliance barrier, yet believed that including parents in child psychotherapy, when working with children who have experienced trauma, enhances parental investment. This is because creating strong working relationships increases parental commitment and motivation for child treatment. Therapists also demonstrated they use the working alliance as a mechanism of change, to mitigate alliance barriers and advance parenting ability in the following areas. First, therapists have the knowledge and expertise to build parental mentalisation capacity, resulting in *“more [parent] capacity to meet the needs of the young person”* (Therapist Bailey). Second, therapists worked with parents to assist them in recognising the child’s undesirable behaviours are trauma-induced responses, for example, “*Dad developed a deeper understanding of why Jason was engaging in such unpleasant and off-putting behaviours”* (Therapist Bailey). Third, therapists proved that the parent–therapist relationship advances parents’ agreeableness in examining their role in the child’s trauma responses. In these circumstances, *“parents are open to feedback, actively involved and understand the impact”* (Therapist Reese, Therapist Nicole, Therapist Maya). The following extract from the self-reflective journal reiterates this finding. Journal entry − 4 October 2023:

I saw in the therapists’ anecdotes that they gently guided parents to see how their actions influenced child trauma healing. In these scenarios, parents were listening and parents were taking responsibility for their behaviours.

While the above-mentioned factors all contribute to decreased parental investment in child psychotherapy, therapists identified parent trauma histories as another obstacle adversely affecting parental support of child trauma-focused psychotherapy.

### Theme 2: therapists’ perspectives on parents’ past trauma

The second prominent theme revealed from the research as a relationship barrier was the parents’ past trauma. This impacted the working alliance as the child’s emotional health is at risk, conflicting with therapy treatment goals and parent trauma reactions manifested as resistant and non-compliant behaviours in the working relationship.

#### Parents’ trauma responses

Most therapists indicated that unprocessed parent trauma manifests in the working alliance. In citing an example, Therapist Maya said, “*The greatest challenge is a parent’s own trauma (be it indirectly related to my client [child’s] trauma or their own trauma)*”. Therapists expressed that working with unresolved parental trauma is extremely difficult for several reasons. To begin with, it *“impact[s] their [the parents’] view of the world”* (Therapist Reece), functioning *(psychopathology mental health, PTSD, attachment difficulties, immature in psychological development*) and parenting capacity. Unprocessed parent trauma also interferes with the formation of a robust parent – therapist alliance, considering parent trauma survivors have trouble with attachment (*attachment issues, attachment problems, impact trust and engagement with the therapist*). Finally, unresolved parental trauma manifests in undesirable behaviours, e.g., aggression, resistance and emotional unavailability in the working alliance. Unresolved parental trauma challenges parent–therapist interactions in multiple ways.

#### The intergenerational cycle of trauma

While many therapists agreed that parents present with unprocessed trauma, six therapists showed that parents continue to transmit intergenerational cycles of trauma to their child *(history of intergenerational physical and verbal abuse, cycle of intergenerational trauma*). Therapists explained that one of the treatment objectives in working with parents who perpetuate trauma cycles is to *“stop trauma cycles*” (Therapist Amelia). However, therapists mentioned that breaking trauma patterns is difficult as parents are “*hard to shift”* (Therapist Rowan). Fundamentally, therapists perceived parent perpetuation of intergenerational trauma cycles as adversely affecting the parent–therapist relationship, since in these situations, parents are a risk to the child’s psychological well-being.

#### Parents’ effect on the child

Most therapists expressed that child maltreatment, by parents who have experienced trauma themselves (*parents cause the trauma, parents can be perpetrators of the trauma, parents are harmful to the child)*, is a common occurrence in their work with children who have experienced trauma. They also observed an association between underlying parent trauma (*traumatic history, own trauma, parent trauma history*) and adverse parenting behaviours, such as *harming the child and/or causing the trauma*. Most importantly, these types of parental behaviours generate/exacerbate the child’s emotional distress, conflict with the therapeutic objective of child trauma recovery and elicit strong therapist emotional responses, disrupting the working alliance.

### Theme 3: therapists’ emotional reactions

The third main theme derived from the data was therapists’ emotions when working with parents. Therapists described parent work as complex, difficult and emotionally challenging for the following reasons. First, therapists have a multifaceted role including treating the child, working with parents (and their associated challenges) and managing the parent–therapist relationship. Second, therapists’ own emotional reactions and associated behaviours triggered by parents’ activities or attitudes have to be managed. Third, in this study, *“parents who had been through trauma themselves*” (Therapist Amelia) presented with a myriad of difficulties, adding a layer of complexity to the working alliance. The two sub-themes resulting from this theme are as follows.

#### Therapists’ emotions when working with parents’ attitudes and behaviours

All 15 therapists explained that parents’ attitudes and behaviours induce strong emotions and reactions. They suggested negative emotions arise in situations where parents are a threat to the child’s emotional safety (*perpetrators of trauma, cause harm, violence*), display uncontained emotions (labelled as therapists as *criticism, demanding, aggression, attack-defence*) and/or when parents engage in behaviours compromising or threatening child treatment *(parents pull their children out of therapy prematurely, threat of pulling them out of therapy*). Though the data indicates parents’ attitudes and behaviours trigger therapists’ emotional reactions, it is the therapist’s responsibility to note, reflect and manage their own emotional reactions and associated behaviours to prevent them from impacting the working alliance.

Six therapists recognised the source (unprocessed trauma) of parents’ maladaptive and treatment-oppositional behaviours. They understood the complex matters causing parents to behave in undesirable ways and recognised *“trauma presents in behaviour rather than deliberate acts of defiance, inattention and restlessness”* (Therapist Reese). Furthermore, therapists with insight into parents’ trauma responses showed less anger and blame towards parents as “*blame is rarely useful and often unfair*” (Therapist Blake), allowing continuity in the working alliance. The following self-reflective journal excerpt echoes this finding. Journal entry − 27 November 2023:

… when therapists were empathetic to parents’ trauma vulnerabilities, parents felt more accepted by therapists, therapists blamed parents less for their adverse trauma behaviours, and parents were more motivated to participate in child treatment.

#### Transference and countertransference in clinical practice

Six therapists identified transference and countertransference in the psychotherapy context when working with parents of children who experienced trauma. Therapists expressed that parents with trauma histories projected/transferred feelings (*anxious, aggressive, critical)* through the therapeutic alliance. Parent traumatic experiences and responses also triggered countertransference emotions in therapists, e.g., Therapist Bailey said, “*Be mindful of transference and countertransference, especially disowned anger*”. In addition, parent trauma experiences can evoke therapists’ own past trauma/unresolved issues, generating therapist transference and countertransference, inadvertently affecting the working relationship.

Though some therapy modalities, e.g., psychodynamic therapy, encourage therapists to work with transference and countertransference emotions, therapists are unable to address these dynamics with parent trauma survivors due to their emotional fragility (*not equipped to cope, immature development, mental health issues*). Given the significance of transference and countertransference when working with this parent cohort, it is essential for therapists to understand and manage their own feelings and reactions. Hence, therapists use good practices such as clinical supervision, peer supervision and professional development (… *make sure you have good supervision, therapist training*) to increase self-awareness, process emotional experiences and transferential feelings and prevent secondary traumatic distress/burnout.

### Theme 4: addressing barriers within the parent–therapist relationship

The fourth theme revealed that therapists are aware of potential alliance barriers when working with parents of children presenting after experiencing a traumatic event and endeavour to address these in treatment plans. In addition, therapists use the parent–therapist relationship as a mechanism to identify and actively address alliance barriers. The following section elaborates on the sub-themes derived from the main theme.

#### Building relationships with parents

All 15 therapists believed a strong parent–therapist relationship makes child therapy more effective when treating children after experiencing trauma. For example, Therapist Thalia expressed, *“If you haven’t got a relationship with the parent, your therapeutic work with the child will be limited”*. Furthermore, in certain therapy modalities such as psychodynamic and family systems therapy, where parents are equally involved in treatment, an enhanced parent – therapist relationship can increase parent involvement and commitment, achieving better treatment outcomes. For example, Therapist Kate said, “*a deeper more loving connection between parent and child and/family system*” and Therapist Gianna added, “*provides the client* (child/parent/family*) relief from symptoms”*. Essentially, all therapists, irrespective of preferred therapy modality, felt a robust parent–therapist alliance promotes “*parent support [of] the child’s engagement in therapy and trust in the therapist which can have a positive effect on therapeutic outcome”* (Therapist Nicole).

#### Parents’ treatment objectives to address relationship barriers

In this study, all therapists who worked inclusively with parents in child trauma psychotherapy were aware of possible alliance barriers (*parents’ own trauma, parents not understanding trauma, parents causing the trauma, level insight into their responsibilities*). Hence, they added relationship-strengthening goals to their treatment plan to accommodate these challenges. First, therapists strived to form a strong partnership with parents. For example, Therapist Gavin said, “*I place relationship before intervention technique”*. Therapist Blake agreed and added, *“Get on the same page and alongside the parents”*. Second, therapists endeavoured to prevent re-traumatising parents (*avoid re-traumatisation, be mindful of not triggering or retraumatising*). Third, therapists attempted to build parent trauma knowledge, and a comprehensive understanding of the child’s trauma needs (*inform about trauma, provide documentation, intergenerational patterns)*. Fourth, therapists managed and supported parents’ trauma needs by “*offer[ing] parents support [for their] own trauma needs; encourage[ing] parents to do their own individual therapy, couples therapy, and or family therapy”* (Therapist Tahlia).

#### The role of being trauma-informed in understanding parents’ emotional needs

The findings showed that numerous parents in this study have experienced adverse childhood events and their emotional responses, e.g., “*PTSD, parental shame, fear, hypervigilance, distress”*, manifested within the working alliance. However, 12 therapists confirmed they are trauma informed and understand how to support parents with their emotional needs. In addition, therapists described being trauma informed as comprising three central components. First, “*knowing the [trauma] research” (*Therapist Tahlia). Second, *“understanding the impact that harmful/overwhelming events and experiences have on a person … affects all aspects of functioning… brain development, altered nervous system, capacity to regulate emotions and ability to fee; safe in their bodies and in the word” (*Therapist Bailey). Third, practicing in a way that develops a *“safe, reliable and consistent therapeutic relationship”* (Therapist Blake), *“avoid[s] re-traumatisation”* (Therapist Thalia) and understands a parent’s *“therapeutic resistance is usually generated by trauma phenomenon rather than willing resistance or poor compliance or treatment behaviour”* (Therapist Gavin). The findings showed that therapists working within a trauma-informed framework can support parents’ trauma reactions to reduce the effect they have on the working relationship.

#### Therapist trauma-focused interventions

The findings suggested that 12 out of 15 therapists have expertise in trauma-focused interventions and are consequently well equipped to support parents when treating children who have experienced trauma. Furthermore, the findings revealed that therapists use the therapeutic alliance as a mechanism with parents to employ a wide range of trauma-focused interventions (*psychodynamic, CBT, solution-focused brief therapy, systems therapy and EMDR)*, “*assess parental functioning and capacity”*, identify areas where parents need greater “*support and information”* (Therapist Nicole), and provide skills to parents enabling them *“to respond more effectively to the young person*” (Therapist Bailey). Therapist Tahlia illustrated that she uses psychoeducation to provide parents with *“an understanding of ‘what’s happened’ NOT what’s wrong with their child* to *support their child in their therapeutic journey*”. Therapist Nicole agreed and added that psychoeducation is a valuable technique to *“develop aspects of parent functioning”* to help parents with *“coping, managing, healing*”. Other therapists described their use of dyadic therapy or family systems therapy to improve relationships, “*shift and repair family systems*, [build] *deeper connections between parent and child, and or family, and breaks cycles of intergenerational trauma”*. Therapists further explained that parents’ functional ability can be improved by “*encouraging … parents to do own individual therapy, couples therapy and or family therapy*” (Therapist Thalia). Largely, the findings revealed that the therapists use the parent–therapist alliance as a medium to contextualise the child’s trauma, identify parent needs and deal with parenting concerns.

## Discussion

The study aimed to explore therapists’ perceptions of the complexities in the parent – therapist relationship when treating children who have experienced trauma. Greater knowledge and understanding of how to manage and support parents in the therapeutic alliance will enhance the working relationship and optimise child treatment success. The research findings revealed that therapists are motivated to form collaborative relationships with parents, as they recognise that parents protect and sustain child treatment success, e.g., parents “*turbo charge[s] child treatment recovery*” (Therapist Gavin). However, therapists consider parent work to be complex and difficult and identified the following alliance barriers: parent investment in trauma-informed child psychotherapy; parents’ past trauma; and difficult therapist emotions. Most importantly, trauma-informed therapists highlighted that alliance barriers can be addressed by making provision for relationship challenges in treatment plans, having knowledge and expertise in identifying and addressing barriers, and developing positive partnerships with parents to maximise child treatment results. All the study’s findings are underpinned by a trauma theoretical framework and confirm that many parents of children who are in psychotherapy after experiencing trauma have themselves experienced trauma.

The findings are mostly in accord with the literature, suggesting that strong parent – therapist alliances strengthen child therapeutic outcomes, while perceived alliance barriers interfere with treatment success. However, unlike working with other types of parents, parents with unprocessed trauma present a significant alliance barrier. Since parent trauma responses, e.g., emotional dysregulation, fear of re-traumatisation and trust issues interfere with child goal attainment and result in difficult parent–therapist interactions. Trauma theory corroborates that parent trauma responses play out as resistant, non-compliant and treatment-interfering behaviours in the parent–therapist partnership. The findings are also consistent with TIC principles, which emphasise that therapists can build strong relationships with parent trauma survivors if they establish a context of trust and safety and encourage collaboration, mutuality and empowerment.

## Clinical implications

The findings are informative and provide insight into therapists’ perspectives of the parent–therapist alliance to enhance understanding of alliance barriers in treatment circumstances. The findings highlight that therapists need to be trauma-informed and apply trauma-informed treatment models when working with parents of children who have experienced trauma, as many parents have experienced trauma themselves. Furthermore, therapists need to have a comprehensive knowledge of trauma theory, understand TIC principles, and recognise parent attitudes and behaviours in the context of trauma. This will help therapists to understand that parents’ non-compliant and resistant behaviours are not deliberate acts of defiance/unwillingness but rather trauma responses. Therapists also need to develop self-awareness and engage in clinical supervision to manage their own difficult emotions regarding parents’ attitudes and behaviours and prevent them from causing alliance barriers. Overall, this new information will enable therapists to manage and support parents more efficiently when they present their child for treatment after they have experienced trauma.

## Limitations

This qualitative study produced rich and in-depth descriptions of therapists’ lived experiences of the parent–therapist relationship when treating children who have experienced trauma, to advance therapists’ understanding of how to better manage parents in the working alliance. A limitation is that the findings cannot be generalised to all parent – therapist relationships when working with children in psychotherapy circumstances. However, the research aim was not generalisability but to deepen therapists’ understanding to assist in developing support mechanisms.

## Preliminary recommendations and future research

Based on the new information, two preliminary recommendations emerged. Child psychotherapists require trauma knowledge and expertise in trauma-specific practices to work with parents when treating children who have experienced trauma. Also, future research studies could be undertaken to compile a set of guidelines for therapists on how to work effectively with parent trauma survivors. Both recommendations would strengthen parents’ investment in child psychotherapy, consequently addressing the broad social issue of childhood trauma. Furthermore, being an exploratory study, additional research studies comprising larger sample sizes and a wider range of states/countries would be valuable to support and validate the findings and preliminary recommendations.

## Conclusion

The exploratory study begins to bridge the gap in the existing literature by describing the tensions and obstacles perceived by therapists in the parent–therapist alliance when working with parents of children who have experienced trauma. It also provides therapists with information on managing treatment situations where they encounter parental treatment non-compliance, parental variable compliance and parent-driven treatment resistance. The new knowledge and understanding of how to better manage and support the parent–therapist relationship places child therapists in a strong position to improve child psychotherapy attendance rates and outcomes, progressing child trauma recovery.

## Data Availability

Data may be made available at the reasonable request of the corresponding author and to the satisfaction of the granting ethics committee.
